# Exploring opportunities for embedding graduate attributes in a first-year undergraduate anatomy course for allied health students

**DOI:** 10.1186/s12909-019-1777-6

**Published:** 2019-09-03

**Authors:** Julian David Pillay, Fazila Ally, Nalini Govender

**Affiliations:** Department of Basic Medical Sciences, Faculty of Health Science, Durban University of Technology, P.O. Box 1334, Durban, 4000 Republic of South Africa

**Keywords:** Team projects, Dissection, video clips, Graduate attributes

## Abstract

**Background:**

There is a growing discontent within the health care industry regarding the state of preparedness of graduates to adequately function in a dynamic work environment. It is therefore required of higher education institutions to equip graduates with skills beyond disciplinary expertise, which would allow them to function optimally in work environments. This study presents a team dissection project that incorporates graduate attributes in an undergraduate first-year anatomy course for the medical orthotics and prosthetics program.

**Method:**

Focus group interviews with students (*n* = 23) were used to demonstrate the achievement of graduate attributes by aligning student perceptions of the dissection project with graduate attributes and indicators thereof.

**Results:**

Students were positive about the effectiveness of the dissection project in enforcing anatomical knowledge; ensuring active engagement with human material; enhancing communication skills and teamwork; and increasing sensitivity towards cultural diversity. These views related largely to those graduate attributes which engage students towards becoming active and reflective learners; creative thinkers; independent and collaborative workers; effective communicators; and culturally and socially aware citizens. Areas of dissatisfaction included challenges with the use of technology for the video preparation; repetition of presentations and large dissection teams.

**Conclusion:**

There is an emerging view that graduate attributes be integrated as early as possible into program curricula so as to become intrinsic in a student’s academic and professional development. Through the expansion of a dissection project forming part of a subject taught very early on in a program’s curriculum, the integration of graduate attributes and discipline-specific competencies are highlighted.

## Background

Employers maintain that emerging graduates, while usually skilled in the subject and career-specific knowledge, lack competencies that would enable them to transform and adapt in their organizations [1–5]. These competencies, popularized as graduate attributes, comprise a set of generic outcomes intended to underpin qualifications and have become increasingly important in higher education [6]. Graduate attributes have become an important factor in the graduate recruitment process [7, 8]. Such attributes typically include interpersonal skills, the ability to work in a team, respect for multiculturalism and diversity, effective communication skills, creative thinking and problem-solving skills [1, 5, 7]. Higher education institutions are mandated to integrate these competencies into the curriculum in order to develop and empower graduates to function in a highly competitive and dynamic work environment [1, 3, 5, 9, 10].

Most universities prioritize the integration of graduate attributes within the curricula. The incorporation, however, appears to be more of an implicit experience that students are expected to acquire in the latter years of a program where vocation-specific practical knowledge is addressed [11]. Edward et al. (2017) describes the acquisition of graduate attributes/work readiness as a “process of development and growth through practice and familiarity” [8]. There is a growing consensus that graduate attributes would become more intrinsic in graduates if integrated into the curricula from the onset of a qualification [12–15]. Such an integration creates an opportunity for foundational disciplines to explore innovative approaches of embedding graduate attributes within the backdrop of discipline-specific needs and challenges.

Team dissection projects can be applied and enhanced to achieve the integration of graduate attributes with discipline-specific content. The involvement of students in teaching relevant topics to the class engages students towards becoming partners in teaching. Team-based learning is reported to reinforce personal responsibility and increase self-confidence thereby allowing for the expansion of interpersonal and collaborative skills [16–18]. More recently, it was shown that first-year medical students identified team-based learning as an effective instructional strategy for understanding anatomy [19]. Team-based learning is, therefore a potential source for reinforcing the development of graduate attributes.

This case study explored the opportunity of integrating graduate attributes in a group of first year students registered for Anatomy I in the Medical Orthotics and Prosthetics program, by introducing a team-based dissection project. The study obtained a synopsis of student perceptions on the effectiveness of the dissection project through focus group interviews. This information was used to connect student perceptions with graduate attributes and contributory indicators thereof. It was hypothesized that modifications to a conventional project can be made to incorporate graduate attributes into courses offered to novice students. Assessment results of the dissection project are presented as evidence of achievement of graduate attributes.

## Methods

This was a qualitative and explorative study, conducted on first year students registered for Anatomy I in the Medical Orthotics and Prosthetics program. In describing the method of the study, the teaching context, as well as the allocation, presentation and assessment of the dissection project are detailed prior to detailing the qualitative and explorative approach of the study.

### Teaching context

The majority of students accessing this program are post-secondary exit students, with Mathematics, Physical Science, Biology and English, as pre-requisite program entry. While English remains a university pre-requisite, almost 60% of all students in the faculty are second-language English speakers. Anatomy-I is a 16-week, prosection-based course, offered in the first semester of year one, that introduces students to Anatomy and covers limb anatomy (Upper limb and Lower limb).

The course includes 1 × 2-h didactic teaching sessions per week and 1 × 3-h practical sessions per week. These didactic sessions include lecture presentations typically using power point slides, incorporating theoretical aspects of the topic and its relevance to clinical application and concluded by an opportunity for questions and further direction. The practical sessions commence with a 10–15 min pre-practical talk, outlining key outcomes of the practical and directing students towards the prosected specimens, anatomical models and bones displayed. During the session, relevant anatomical models, plastinated and prosected (pre-dissected) specimens and bones are displayed in the laboratory as the learning resources. The session is concluded by a post-practical discussion that reviews the outcomes of the session and provides further direction. These sessions are facilitated by the lecturer, with a demonstrator assisting in the learning experience. For ongoing reference, power point presentations, tutorials and other relevant resources are available to students through the electronic platform of the institution at the beginning of the course.

Formal assessment of the course includes a two-hour theory test and a 90-min practical test (conducted between weeks 8 and 10, each constituting 15% of the final mark); a team project (conducted during weeks 2 to 6, constituting 10% of the final mark); 1 × 3-h theory examination and a 90-min practical examination (conducted between weeks 15 and 16, each constituting 30% of the final mark). An average of 50%, with a minimum of 40% in each assessment is required for successful completion of course.

### Allocation of project

All registered students (*n* = 34) were randomly allocated into teams of four to five. Each team was allocated a specific topic based on four of twelve key dissection areas of limb anatomy viz. the brachial plexus, the anterior compartment of the forearm, the anterior compartment of the thigh, and the posterior compartment of the thigh. Other keys areas of the upper limb and lower limb were taught via conventional lectures and the use of prosected specimens during practical sessions.

As there were no lectures provided for the topics covered by the dissection project, students were allowed the lecture time to further work on their dissections and video presentation, as well as obtain/review relevant information.

### Presentation of the project

Teams were required to practically demonstrate the selected dissected region and supplement it with a self-produced video clip, followed by a question and answer session. The practical demonstration served to highlight relevant anatomical structures and their relationships as part of the dissection experience, including the demonstration of theoretical knowledge of the area. The video clip, recorded through any commonly used mobile device such as an iPhone or iPad, displayed the dissection and incorporated more in-depth explanations and supplementary resources. This was followed by a short session allowing for questions and answers. Members of the team were required to respond to questions about aspects that related to the area dissected and presented. The presentation of the dissection and video was conducted in a theatre-type lecture venue so as to allow easy and clear viewing by all students during group presentations. The dissected specimens were subsequently displayed in the laboratory for closer viewing and independent learning by students.

### Assessment of the project

The teams were assessed (by the lecturer) on three elements of the dissection project as summarized, with each element and sub-elements therein, having an equal weighting:

#### Demonstration of the dissection

- Aesthetic appearance of project
- Highlighting of relevant structures and their relationships
- Ability to link theoretical knowledge to the dissection

#### Video clip presentation

- Creativity of presentation
- Competency in the use of technology
- Engagement with dissection

#### Question-and-answer session

- Appropriateness of information presented/responses
- Ability to explain coherently/logically
- Participation of team members

### Focus group interviews to obtain student perceptions

Student perceptions of the dissection project were obtained through focus group interviews of 30–45 min duration. The focus group interviews were conducted in the departmental boardroom by an independent and experienced external facilitator. An external facilitator was used to limit the bias of the lecturer requesting feedback from students on a teaching event facilitated by the same lecturer. To further encourage honest feedback, the interviews took place after final course assessments were completed so that students felt comfortable in their participation/non-participation in the interviews.

Students were invited to participate in the focus group interviews through an internal email forwarded by the external facilitator of the focus group interviews. The facilitator outlined her background as a psychologist and her experience in conducting interviews and further went on to outline the purpose of the study. Based on the voluntary nature of the study, some students chose not to participate. Notwithstanding this, the focus group interviews were used as an opportunity to establish some awareness of student perceptions of the project and to thereby help engage academics in reflective practice using student feedback.

Students were randomly allocated into four focus groups, each comprising 5–6 students. To ensure heterogeneity of perspectives, participants were allocated to focus groups independent of the project group that they were assigned to.

The focus group interviews were directed around three key aspects, each including one or more initiating questions with the latitude to expand into further questions/discussions as identified by the interviewer:

#### The project

- What do you think were the reasons for engaging you, as a class, in such a project?
- What did you enjoy about the project?
- Were there any aspects of the project that you did not enjoy?

#### Subject content

- Over and above the support provided by the lecturer on these topics, how have the different projects collectively enhanced your knowledge and application of the subject content?
- Do you think that such projects provide an alternative method of teaching and learning to the typical class lecture?

#### Future recommendations

- What areas for improvement of this learning experience would you suggest?

### Data analyses

The attainment of graduate attributes was assessed using the perceptions of students as per the focus group analyses. The relevant graduate attributes were applied as themes while the sub-themes were identified as indicators emerging from the data that related to the specific themes. These graduate attributes, categorized as themes, included active and reflective learning; creative thinking; effective communication and social awareness.

All focus group interviews were audio-recorded and transcribed by the external facilitator and coded by the researchers. Thematic analyses were used to analyze the transcripts using the NVivo, version 10, qualitative data analysis software (QSR International Pty Ltd., Melbourne, Australia).

The outcome of the thematic analyses are presented as evidence of achievement of the graduate attributes. Class performance in each of the nine assessment criteria are presented as means and standard deviations.

## Results

### Focus group interviews

Four focus group interviews were conducted, with 23 students of a class of 34, yielding a 68% response rate. The focus group explored various aspects on the project with particular reference to participation in the dissection project and engagement with course content. Written transcripts arising from the focus group discussions were linked to selected graduate attributes, identified as themes.

A range of activities that served as indicators in achieving the desired graduate attribute were identified as sub-themes, extrapolated through student perceptions (Table 1). For example, the students’ ability to expose the relevant structures in their respective projects (to explain their relationships within the specimen and to link the ability to integrate appropriate theoretical knowledge with the dissected specimen) highlighted their capacity to function as “*active and reflective learners*” (Table 1: quotes 4,5,6,7). Working as part of a team to complete the dissection project also helped the students to develop skills which would allow them to become “*creative thinkers that worked individually and collaboratively within the team*”.

**Table 1:** Student perceptions in relation to the graduate attributes and contributing indicators

Graduate attributes (Themes)	Indicators of graduate attributes (Sub-themes)	Student perceptions (quotations)
Active and reflective learners; creative thinkers that work independently and collaboratively	Interactive collaboration	^*1*^*“to learn from each other* and *to teach others”* ^*2*^ *“helped us to tolerate and understand each other”* ^a^ ^*3*^ *“Getting the group members together was a problem and this delayed the making of the video”* ^a^
	Learning through practice	^*4*^ “*the actual dissecting was interesting because books do not explain as good as you doing it yourself”* ^*5*^ *“By dissecting ourselves, we felt more involved and engaged with the subject”*
	Independent, reflective learning	^*6*^ *″Enjoyed looking for information by ourselves rather than depending on the lecturer”* ^*7*^ *“Doing it yourself you tend to remember it more”* ^*8*^ *“To help us learn an alternative method of learning and teaching”*
Effective communicators	Competency in responses	^*9*^ *″Empowering us with presentation skills”* ^*10*^ *″Some teams extensively researched their topics, and therefore increased our knowledge of the topic”* ^*11*^ *″Some teams presented with confidence and helped us gain more knowledge”*
	Participation of team members	^*12*^ *“Helped us in the planning of ideas together”* ^*13*^ *“Different individual skills were seen – confident students presented while the shy students dissected”* ^*3*^ *“Getting the group members together was a problem and this delayed the making of the video”* ^a^
	Use of technology in the production of the video	^*14*^ *“Gave us the opportunity to work with computers”* ^*15*^ *“Gain experience in developing a formal video- learning a new skill”* ^a^ ^*16*^ *“Video presentation is better than the lecturing method because it is easier to remember when you see it”* ^*17*^ “*The technical challenges of making the video was time consuming, frustrating and tiring”* ^a^ ^*18*^ *″We seemed to lack the specific computer skills required for making the videos”* ^a^
Social awareness	Social sensitivity	^*2*^ *“Helped us to tolerate and understand each other”* ^a^ ^*19*^ “*preparing us to work with other people in later life in hospitals,* etc.”
	Confidence enhancement	^*20*^ *“To boost our self-esteem and confidence”* ^*21*^ *″Stimulated us to participate even though we would not have, otherwise”*
	Technological expansion	^*15*^ “*Gain experience in developing a formal video – learning a new skill”* ^a^ ^*17*^ “*The technical challenges of making the video was time consuming, frustrating and tiring”* ^a^ ^*18*^ *″We seemed to lack the specific computer skills required for making the videos”* ^a^ ^*22*^ *“Developing a video clip was a good experience and we also became actors, lecturers and graduates”* ^*23*^ *“Lecturers aim was trying to improve our learning skills and to introduce us to new learning skills”* ^*24*^ *“I think doing a video was irrelevant to our study of anatomy”*
	Environmental cognizance	^*25*^ *“To teach us to follow correct procedures in the dissection of human parts”*

^a^ *indicates quotes applicable to multiple sub-themes/themes*

Students also expressed an appreciation of this teamwork through various sentiments (Table 1: quotes 1, 2 and 19). In addition, many students indicated that the project activity boosted their confidence and technological understanding, as well as allowed them to be socially aware of their peers (Table 1: quotes 2, 19–23).

Despite the evidence that the project activity supports a high level of student enjoyment and knowledge application, students highlighted a few concerns and limitations, as presented (Table 1: quotes 3, 17, 18, 24). Some areas of concerns included the repetition of class presentations (i.e. two presentations per topic) which was reported to be tiring and boring to some students, who felt that information presented by two teams was laborious. Others felt that the teams were too large and should be made smaller in the future. Technical difficulties encountered in the preparation of the video repeatedly emerged as a challenge to students (Table 1: quotes 17, 18).

### Team performance in the project

**Fig. 1:**
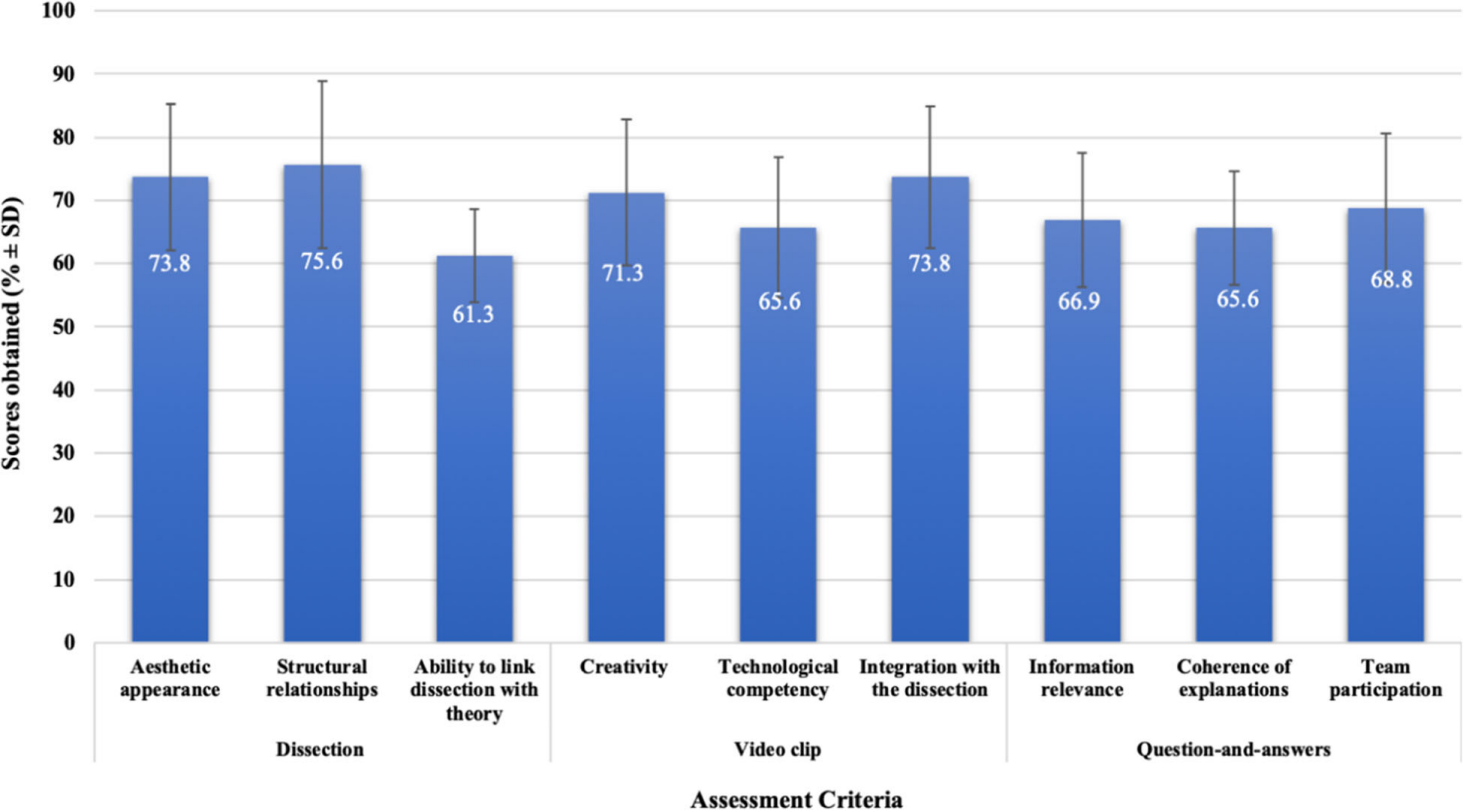
Project Performance (% ± SD)

A synopsis of student performance in the project, based on the three elements of the project and the assessment criteria in each element (highlighted in the methodology) is shown (Fig. 1).

For the dissection, an average score of 74 and 76% were obtained for assessment criteria related to aesthetic appearance and structural relationships respectively. This is suggestive of good dissecting skills as a team. However, the average score of 61%, when assessed on the ability to link the dissection with theory, indicates a possible disjuncture between theoretical knowledge and practical demonstration of structures dissected. This is further highlighted in the question-and-answer component which directly relates to content knowledge, for which teams scored lower than most of the other assessment criteria.

With regard to the development of the video clips, teams scored between 71 and 74% for creativity and integration with the dissection respectively. The high scores obtained for aesthetic appearance (dissection) and both creativity and integration with the dissection (video-clip) is suggestive of the teams’ ability to perform well in the more skills-based activities of the project. Technological abilities scored lower than most other assessment criteria, and an aspect similarly emerging as a challenge in the focus group discussions.

## Discussion

Over the last two decades, the concept of graduate attributes has gained increasing emphasis within the higher education sector [1, 15]. Although the attainment of graduate attributes is traditionally viewed as an exit level outcome, recent emphasis has been placed on integrating these attributes from inception to completion of a program [8, 20]. Consequently, graduate attributes need to be embedded in the curriculum at a subject level [1, 9, 20–22] and the pedagogical approaches utilized need to actively engage students to encourage the explicit development of these attributes in a coherent and structured manner [5, 8, 12, 14, 15, 21].

Academics have highlighted a number of barriers that hamper the integration of graduate attributes into curricula. These include the lack of additional teaching and assessment time, limited resources for innovative approaches, little or no support for varied activities and limitations around student readiness for such engagement [23]. However, assessment is an aspect of the learning experience that has been identified as an opportune site to incorporate the development of graduate attributes into the learning experience [24]. Consequently the alignment of assessment with the demonstration of graduate attributes has been touted as a way forward [24].

This team-based dissection project was a multi-faceted’ approach to afford the students the opportunity to practice and develop graduate attributes. The dissection project required the students to research the area to be dissected, devise a dissection strategy to clearly demonstrate features of the dissected regions, creatively communicate the project in an oral seminar to an audience of peers and the examiner, and justify their chosen strategy through coherent responses in the question and answer segment. The project presented in this paper highlights the graduate attributes that have been integrated into a dissection project and demonstrates the extent to which some of these have been attained, and student perceptions thereof.

### Effective communication skills

In creating and presenting the dissected project and video presentation, communication skills, teamwork and the use of technology were identified as important, by students consistently across the teams (Table 1: quotes 1, 2, 12, 19). Effective communication skills and teamwork have been reported as key attributes in the professional life of health care practitioners [5, 9, 12, 25]. In a systematic review of team-based learning in health professions education, Reimschisel et al. (2017) reported that team-based learning was effective in teaching skills such as teamwork, effective communication, problem solving and conflict resolution [26]. This was corroborated by Currey et al. (2015) and Hahn and Ryu (2017) who reported a positive link between team-based learning and the development of graduate attributes [29, 30].

### Awareness and sensitivity of cultural diversity

Further to the indication by students of the evidently strong teamwork needed and enhanced by the dissection project, some students reported that working in teams allowed them opportunities to understand and be sensitive towards team members from diverse backgrounds (Table 1: quotes 2, 13, 19). This particularly relates to the awareness and sensitivity of cultural diversity as a graduate attribute and an attribute important for health professionals who are required to engage with patients and colleagues as part of local and international teams [4, 7, 9, 26].

### Self-directed learning

Self-directed learning, through the evaluation of information and resources, emerged as an outcome of the project that students highlighted (Table 1: quotes 4–7). Students emphasized the active engagement with subject content that facilitated and ensured a more self-directed approach. This outcome highlights key elements of graduate attributes related to active and reflective learning, the importance of which several studies have reiterated, in the daily activities of all allied health professionals [29–31].

### Future considerations

A few modifications and considerations should be applied to enhance the project experience:

- At the outset, a clear outline should be provided to students as to why such a project which particularly emphasizes the graduate attributes, is to be embarked on;
- Provision of an assessment rubric to teams prior to the project and details of team score as per the rubric;
- The link between specific project outcomes and the graduate attributes should be made explicit.
- Provision of a suitable space and appropriate software/device for video recordings;
- Suitable training for video recording be provided.

### Limitations and recommendations

The findings of this study should be construed in consideration of some limitations, namely: the research included data obtained from a single source only, which was first-year students at a single institution and the method of data collection was via focus group discussions. However, the strategy used in this study may be applicable across disciplines, as graduate attributes are essential to all fields and levels of study. Future research should consider supplementing focus group data with a structured survey. Additionally, stratification of the results by demographic differences and its influence on the extent of development and perceptions of graduate attributes may provide more insightful findings. Further research is required to include the views of all stakeholders (academics, students and demonstrators) in the teaching and learning environment, as well as a more diverse representation of students from different programs and institutions. Moreover, the use of a single evaluator in our study may be considered a limitation due to possible bias.

## Conclusion

Transformation in higher education, largely driven by policy, warrants a more interactive and self-directed learning environment. A significant gap appears to exist between the desired graduate attributes and their development and integration in the curriculum. Therefore, it is necessary to revisit curriculum design to incorporate learning activities and assessment tasks that would afford students opportunities to develop and practice the desired graduate attributes. This provides a platform for the integration of graduate attributes into discipline-specific competencies which, if applied early in the academic trajectory of the student, would support a more internalized and deep-rooted entrenchment of the graduate attributes.

## Data Availability

The datasets used and/or analysed during the current study are available from the corresponding author on reasonable request.
